# Preoperative Evaluation of Risk of Ovarian Malignancy Algorithm Index in Prediction of Malignancy of Adnexal Masses

**DOI:** 10.5812/ircmj.17185

**Published:** 2014-06-05

**Authors:** Farah Farzaneh, Zahra Honarvar, Mansoore Yaraghi, Mehdi Yaseri, Maliheh Arab, Maryamsadat Hosseini, Tahereh Ashrafgangoi

**Affiliations:** 1 Department of Gynecology-Oncology, Shahid Beheshti University of Medical Sciences, Tehran, IR Iran; 2 Department of Statistics and Epidemiology, Tehran University of Medical Sciences, Tehran, IR Iran

**Keywords:** CA-125 Antigen, Diagnosis, Ovarian epithelial cancer, Tumor Markers, Biological

## Abstract

**Background:**

Differentiation between benign and malignant ovarian neoplasms is essential to create a system for patient referrals.

**Objectives:**

The aim of the present prospective trial was to analyze the value of the risk of ovarian malignancy algorithm (ROMA) in prediction of adnexal masses malignancy in pre- and post-menopause women before operation.

**Materials and Methods:**

Preoperative serum samples were tested for CA125 and HE4 using fully automated methods (Abbott architect) and gained best cutoff. The ROMA index was analyzed in 99 patients (including 68 pre-menopause and 31 menopause) with adnexal masses referred to Imam Hossein Hospital/Tehran/Iran and had been scheduled for operation. The pathological results showed 43 cases (22 menopause) with malignant adnexal masses and 56 cases (9 menopauses) with benign adnexal masses. Demographical data, clinical symptoms and the ROMA index were separately analyzed and contrasted in benign and malignant in both menopause and pre-menopause patients.

**Results:**

The only significant difference was the older age of the malignant group vs. benign group (P = 0.001) regarding demographic findings. As concerns the clinical symptoms, presence of abdominal discomfort in pre-diagnosis period was the only significant parameter in malignant group (P = 0.001). Additionally, data analysis of patients as a total group showed that specificity (96.4%), positive predictive value (PPV) (94.1%), area under the curve (AUC) (0.907), and diagnostic accuracy (DA) (86.9%) of the ROMA were higher than HE4 (91.1%, 85.7%, 0.857 and 81.8%. respectively) and CA125 (87.9%, 67.3%, 0.828 and 75.8%, respectively) alone. Besides, negative predictive value (NPV) (86.4%) and sensitivity (86.1%) of CA125 were higher than HE4 (79.7% and 69.8%, respectively). In contrast, specificity of HE4 (91.1%) was higher than CA125 (67.9%). Data analysis of patients as two groups (pre and post menopause groups) showed the same results.

**Conclusions:**

Specificity, DA and AUC of the ROMA were higher than HE4 and CA125 taken separately.

## 1. Background

Ovarian cancer is the seventh most common malignancy in American women and the eighth in Iranian women (1). Among all gynecologic cancers, ovarian malignancy has attracted the most clinical debate, since it has the highest fatality to-case ratio of gynecologic malignancies, also often asymptomatic and in approximately three forth of cases is diagnosed in advanced stage and accompanied by metastasis (2).

In management of ovarian cancer, the most critical point is initial surgery, which includes thorough surgical staging in early stages and optimal debulking in advanced stages. In the United States of America, between 169,000 and 289,000 women are hospitalized with an ovarian cyst or pelvic mass annually. About 5-10 percent of total American women undergo surgery because of ovarian mass during their life (3). The National Institute of Health (NIH) indicated that 13-21 percent of all patients with ovarian masses have invasive epithelial ovarian cancer (4). Prediction of malignancy in adnexal mass before operation is very important, so that the patient could be referred to well-equipped centers and undergo surgery by gyneco-oncologists in case of high probability of malignancy. In this way, the patient might not need reoperation, which itself increases morbidity. 

Therefore, researchers have recently tried to create a method with acceptable sensitivity and specificity by which they can predict the possibility of malignancy in ovarian masses before operation. Serum level of CA125 is commonly measured to predict malignancy probability in women with pelvic masses. However, measuring CA125 has some limitations. This biomarker is increased only in less than 50% of early stage ovarian cancers. Most gynecology and some non-gynecology disorders, whether pre-menopause or post-menopause, may increase serum level of CA125, which decreases sensitivity and specificity of this test in diagnosis of epithelial ovarian cancer (5). For this reason, researchers used both sonographic findings and CA125 level as risk of malignancy index (RMI). Application of CA125 and pelvic sonography together increases the sensitivity and specificity in diagnosis of ovarian cancer (6). RMI has also limitations. For instance, sonographic findings are dependent on sonographer’s care and experience; moreover, clinical evaluation of pelvic mass includes computed tomography (CT) and magnetic resonance imaging (MRI), whose results are lacking in RMI.

In the recent decade, ROMA index has been introduced to distinguish benign masses from malignant masses, which is a formula including two biomarkers, HE4 and CA125. HE4 is a new biomarker, which might be increased in serum of patients with epithelial ovarian cancer based on some studies. According to these studies (7, 8), HE4 is more sensitive than other examined biomarkers and even CA125; and it is believed that measuring both CA125 and HE4 as ROMA index may be applied for women with ovarian cancer. Such method has not been definitely approved yet; besides, some studies have reported the opposite (9, 10).

## 2. Objectives

The aim of the present study was to measure the blood level of two biomarkers to evaluate the efficacy of ROMA method in diagnosis of adnexal masses before operation in women with pelvic masses hospitalized in Imam Hossein Hospital.

## 3. Materials and Methods

This was a prospective trial conducted at the Department of Obstetrics and Gynecology of Imam Hossein Hospital (Shahid Beheshti University, Tehran, Iran) and is the first phase of an ongoing study on the evaluation of the ROMA index value to predict malignant epithelial ovarian cancer, prognosis and survival of patients. Patients with the following criteria entered the study; their menarche had been at least one year before, they had adnexal masses, and they were scheduled for a surgery. Before collecting biologic and surgical samples, all required information about research purpose and its method was given to the patients in writing and verbally; then, in case of their written permission and agreement, preliminary data form was completed. The form included patients’ demographic information such as age, menarche age, parity, gravidity, menopause age, contraceptive method, breast feeding duration, as well as information about menstrual habits and their symptoms before admission. Among all patients with ovarian masses who visited Imam Hossein Hospital from March 2012 to March 2013, 99 were studied (56 with benign pathology and 43 with epithelial ovarian cancer malignant masses).

In these patients, 5 mL blood sample was taken 30 minutes before the operation to measure HE4 and CA125. The blood sample clotted in room temperature in 10 minutes and was centrifuged in 30 minutes and serum and plasma were separated. The serum level of CA125 was measured using the Architect CA125II assay (Abbott Diagnostics, Abbott Park, IL); and serum HE4 levels were determined using the HE4 EIA assay (Fujirebio Diagnostics Inc.). 

Patients were divided into two groups based on pathological findings, women with benign ovarian masses and those with epithelial ovarian cancer. At first, the two groups were compared regarding demographic information, clinical symptoms, and laboratory results. Then the data was reanalyzed in two subgroups of menopause and pre-menopause women. Women were considered as menopause if they had their last period at least one year before or had been approved to be menopause by laboratory test. After recording CA125 and HE4 levels of all patients, the ROMA index was calculated and registered according to the following formulae:

Predictive Index:

Premenopausal: (PI): -12 + 2/38 × LN (HE4) + 0/0626 × LN (CA125)

Postmenopausal: (PI): -8/09 + 1/04 × LN (HE4) + 0/732 × LN (CA125)

The ROMA Index:

ROMA (%) = e^PI^/ (1 + e^PI^) × 100

Considering that the ROMA cutoff is different in pre-menopause and post-menopause women, ROMA was analyzed first in all patients and then separately in the two subgroups of pre-menopause and post-menopause patients.

### 3.1. Statistical Methods

To describe data, frequency (percent), mean ± SD, 95% confidence interval, median, and range were measured. To evaluate the difference between the two groups in baseline, Chi-Square and Mann-Whitney were used. To evaluate the performance of the ROMA index in predicting epithelial ovarian cancer, Receive Operating Curve (ROC) was used. Then the best cutoff point was obtained by Youden index from this curve, considering the patients’ menopausal status, and sensitivity, specificity, positive predicted value, negative predicted value, likelihood ratio, diagnostic accuracy, diagnostic Odds and Cohen's kappa index with their 95% confidence interval for this cutoff point were all evaluated. P-value less than 0.05 was considered statistically significant. All statistical analyses were performed by SPSS software (Version 21.0, IBM Co., and Chicago IL).

## 4. Results

**Figure 1.**
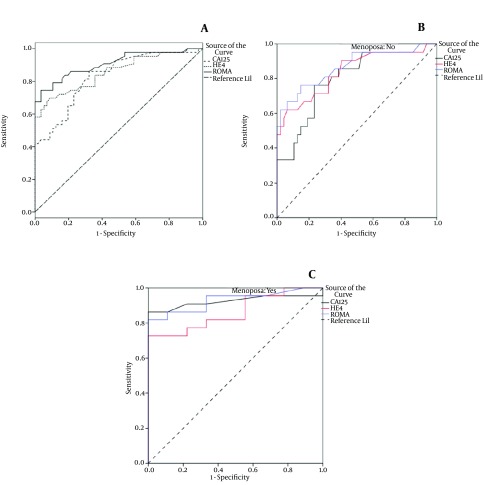
AUC in Patients (A), pre-menopause group (B), and post-menopause group (C).

In general, during the one year of the present study, among 106 women with ovarian masses who were scheduled for laparotomy, 100 women entered the study. Frozen section results were considered; while, 6 were excluded from the study, due to non-epithelial origin of their masses, and one patient was not interested to enter the study. Hence, according to pathological results, 56 had benign masses and 43 had epithelial ovarian cancers. In the present study, the benign group were teratoma (n = 12), endometriomas (n = 10), fibroma (n = 2), mucinous cyst adenoma (n = 10), serous cyst adenoma (n = 12) and simple cyst (n = 6). The malignant group were serous cyst adenocarcinoma (n = 28), mucinous cyst adenocarcinoma (n = 8), endometrioid carcinoma (n = 4) and clear cell carcinoma (n = 3). Two women with metastatic malignant pathology, one metastatic adenocarcinoma of gastrointestinal, one metastatic adenocarcinoma of breast were excluded as well as 3 cases of granulosa cell tumors and 1 case of germ cell tumor due to their non-epithelial origin. Then, demographic data, clinical symptoms, and the ROMA index were compared between the two groups. The mean age of patients was 44, with a significant difference between patients with benign masses and those with malignant masses (39 years vs. 51 years, P = 0.001). The demographic factors were presented in Table 1. The mean age for menarche (11.9 years), the average of parity (2.6 deliveries), gravity (2.8 pregnancies), history of OCP (Oral Contraceptive Pill) consumption, and breast feeding duration did not have a significant difference between control and case groups. Demographic data in pre-menopause and post-menopause were analyzed separately (data are not given). In that relation, from 31 post-menopause patients, 22 had malignant masses, and from 68 pre-menopause patients 21 had malignant masses, which was not statistically significant. Clinical symptoms were compared between control and case groups, as given in Table 2. From clinical symptoms, only abdominal distention (P = 0.001) was significantly higher in case group. Then, clinical symptoms were re-analyzed in pre-menopause and post-menopause patients (data are not given); only abdominal distention was significantly higher in malignant pre-menopause patients (P = 0.001). As concerns the comparative analysis of biochemistry, level of CA125, HE4, the ROMA index and AUC with 95% CI between the case and control groups were shown in Tables 3 and 5. The same comparison was also performed in the two subgroups of pre-menopause and post-menopause (Table 4, Figure 1 A, B and C). The data was first generally analyzed without dividing patients into pre-menopause and post-menopause groups. The best cutoff point was obtained by the Youden index. On that basis, CA125 sensitivity in cutoff 22.5 for diagnosis of EOC was 86.1%, its specificity was 67.9%, PPV was 67.3%, NPV was 86.4%, and AUC was 0.828. HE4 sensitivity in cutoff 73 for diagnosis of EOC was 69.8%, its specificity was 91.1%, PPV and NPV were 85.7% and 79.7% respectively, and AUC was 0.857. The ROMA index sensitivity in cutoff 18.3 for diagnosis of EOC was 74.4%, its specificity was 96.4%, PPV and NPV were 94.1% and 83.1% respectively, and AUC was 0.907. In the next stage, data was re-analyzed by dividing patients into pre-menopause and post-menopause groups with the following results: In 68 patients of pre-menopause group, CA125 sensitivity in cutoff 35 for diagnosis of EOC was 76.2%, its specificity was 72.3%, PPV was 55.2%, NPV was 87.2%, and AUC was 0.81. HE4 sensitivity in cutoff 75 for diagnosis of EOC was 57.1%, its specificity was 95.7%, PPV and NPV were 85.7% and 83.3% respectively, and AUC was 0.839. The ROMA index sensitivity in cutoff 11.5 for diagnosis of EOC was 76.2%, its specificity was 85.1%, PPV and NPV were 69.6% and 88.9% respectively, and AUC was 0.868. In 31 patients of post-menopause group, CA125 sensitivity in cutoff 25 for diagnosis of EOC was 86.4%, its specificity was 100%, PPV was 100%, NPV was 75%, and AUC was 0.924. HE4 sensitivity in cutoff 100 for diagnosis of EOC was 72.7%, its specificity was 100%, PPV and NPV were 100% and 60% respectively, and AUC was 0.864. The ROMA index sensitivity in cutoff 25.5 for diagnosis of EOC was 81.8%, its specificity was 100%, PPV and NPV were 100% and 69.2% respectively, and AUC was 0.929. Total analysis of data without dividing patients into pre-menopause and post-menopause groups showed the diagnostic accuracy of CA125 as 75.8%, HE4 as 81.8%, and the ROMA index as 86.9%. Analysis of data with dividing patients into pre-menopause and post-menopause groups showed the diagnostic accuracy of CA125, HE4 and the ROMA index as 73.5%, 83.8% and 82.4%, respectively in pre-menopause group, and 90.3%, 80.7% and 87.1% respectively in post-menopause group. From 43 patients of malignant group, 4 cases (9.3%) were in stage 1, 8 (18.6%) in stage 2, 22 (51.2%) in stage 3 and 9 (20.9%) in stage 4 (Table 6). Considering the number of cases (43), comparison of the value of the ROMA with stage of the malignancy was performed between the early stage group (ESG) (including stages 1 and 2) and the advanced stage group (ASG) (including stages 3 and 4). The ROMA sensitivity in the ESG and the ASG were 75% and 74.2% respectively, and its specificity in both of these two groups was identical (96.4%). PPV of ROMA in ASG (92%) was higher than that of ESG (81.8%). NPV of ROMA in ESG (94.7%) was higher than that of ASG (87.1%). DA of ROMA in ESG (92.7%) was higher than that of ASG (88.5%) (Table 7).

**Table 1.** Demographic Data of Patients ^a,b^

	Total	Group		P Value
		Benign	Malignant	
**Age, y**				0.001 ^c^
	44 ± 16	39 ± 14	51 ± 16	
	45 (17 - 79)	37 (17 - 76)	51 (18 - 79)	
**Menarche age, y**				0.290 ^c^
	11.9 ± 1.2	12 ± 1.3	11.7 ± 1.2	
	12 (9 - 14)	12 (9 - 14)	12 (9 - 14)	
**Parity**				0.465 ^c^
	2.6 ± 2.5	2.4 ± 2.4	2.8 ± 2.7	
	2 (0 - 12)	2 (0 - 9)	2 (0 - 12)	
**Gravity**				0.586 ^c^
	2.8 ± 2.6	2.7 ± 2.6	3 ± 2.7	
	2 (0 - 12)	2 (0 - 10)	3 (0 - 12)	
**Menopause**	31 (31.3)	9 (16.1)	22 (51.2)	< 0.001 ^d^
**Menopause age, y**				0.567 ^c^
	50 ± 3.7	50 ± 2.6	50 ± 4.1	
	50 (40 - 55)	50 (45 - 54)	50.5 (40 - 55)	
**OCP, y**				0.187 ^c^
No	77 (77.8)	41 (73.2)	36 (83.7)	
1	14 (14.1)	9 (16.1)	5 (11.6)	
1-5	5 (5.1)	3 (5.4)	2 (4.7)	
> 5	3 (3.0)	3 (5.4)	0 (0)	
**Breast feeding**				0.772 ^c^
0	27 (27.3)	16 (28.6)	11 (25.6)	
1	19 (19.2)	12 (21.4)	7 (16.3)	
2	13 (13.1)	5 (8.9)	8 (18.6)	
3	13 (13.1)	8 (14.3)	5 (11.6)	
> 3	27 (27.3)	15 (26.8)	12 (27.9)	

^a^ Abbreviation: OCP, oral contraceptive pill.

^b^ Data are presented as mean ± SD, Median (range) or No. (%).

^c^ Based on Mann-Whitney test.

^d^ Based on Chi-square test.

**Table 2.** Clinical Symptoms of Patients ^a^

	Total	Group		P Value
		Benign	Malignant	
**VB CC**	23 (23.2)	13 (23.2)	10 (23.3)	0.966 ^b^
**Pain CC**	53 (53.5)	32 (57.1)	21 (48.8)	0.411^b^
**Distension CC**	25 (25.3)	7 (12.5)	18 (41.9)	0.001 ^b^
**Weight Loss CC**	3 (3.0)	0 (.0)	3 (7.0)	0.079 ^c^

^a^ Data are presented as No. (%).

^b^ Based on Chi-square test.

^c^ Based on Fisher exact test.

**Table 3.** Sensitivity, Specificity, Positive and Negative Predictive Value of the ROMA Index, HE4, CA125 in Patients ^a,b^

	Variables		
	CA125 (≥ 22.5)	HE4 (≥ 73)	ROMA (≥ 18.3)
**True Positive**	37	30	32
**False Positive**	18	5	2
**False Negative**	6	13	11
**True Negative**	38	51	54
**Sensitivity (95% CI)**	86.1 (72.74, 93.44)	69.8 (54.89, 81.4)	74.4 (59.76, 85.07)
**Specificity (95% CI)**	67.9 (54.82, 78.6)	91.1 (80.74, 96.13)	96.4 (87.88, 99.02)
**Positive Predictive Value (95% CI)**	67.3 (54.1, 78.19)	85.7 (70.62, 93.74)	94.1 (80.91, 98.37)
**Negative Predictive Value (95% CI)**	86.4 (73.29, 93.6)	79.7 (68.29, 87.73)	83.1 (72.18, 90.28)
**Diagnostic Accuracy (95% CI)**	75.8 (66.46, 83.13)	81.8 (73.08, 88.18)	86.9 (78.82, 92.16)
**Likelihood ratio of a Positive Test (95% CI)**	2.7 (2.38 - 3.011)	7.8 (5.133 - 11.9)	20.8 (7.658 - 56.7)
**Likelihood ratio of a Negative Test (95% CI)**	0.21 (0.1447 - 0.2921)	0.33 (0.2844 - 0.3874)	0.27 (0.2217 - 0.3175)
**Diagnostic Odds (95% CI)**	13 (4.653 - 36.43)	23.5 (7.636 - 72.55)	78.6 (16.36 - 377.1)
**Cohen's kappa (Unweighted) (95% CI)**	0.5 (0.3308 - 0.7134)	0.6 (0.4276 - 0.816)	0.7 (0.5327 - 0.9195)

^a^ Abbreviations: CI, confidence interval; ROMA, risk of ovarian malignancy algorithm.

^b^ Data are presented as Median (Range).

**Table 4.** Sensitivity, Specificity, Positive and Negative Predictive Values of the ROMA Index, HE4, CA125 in Two Subgroups of Pre-menopause and Post-menopause Patients ^a,b^

	Under Menopause Age (n = 68)			Above Menopause Age (n = 31)		
	CA125 (≥ 35)	HE4 (≥ 75)	ROMA (≥ 11.5)	CA125 (≥ 25)	HE4 (≥ 100)	ROMA (≥ 25.5)
**True Positive**	16	12	16	19	16	18
**False Positive**	13	2	7	0	0	0
**False Negative**	5	9	5	3	6	4
**True Negative**	34	45	40	9	9	9
**Sensitivity (95% CI)**	76.2 (55 - 89)	57.1 (36 - 75)	76.2 (55 - 89)	86.4 (66 - 95)	72.7 (51 - 86)	81.8 (61 - 92)
**Specificity (95% CI)**	72.3 (58 - 83)	95.7 (86, 99)	85.1 (72 - 93)	100 (70 - 100)	100 (70 - 100)	100 (70 - 100)
**Positive Predictive Value (95% CI)**	55.2 (38 - 72)	85.7 (60.- 96)	69.6 (49 - 84)	100 (83 - 100)	100 (80 - 100)	100 (82 - 100)
**Negative Predictive Value (95% CI)**	87.2 (73 - 94)	83.3 (7 - 901)	88.9 (76 - 96)	75 (46 - 91)	60 (35 - 80)	69.2 (42 - 87)
**Diagnostic Accuracy (95% CI)**	73.5 (62 - 83)	83.8 (73 - 91)	82.4 (71 - 90)	90.3 (75 - 96)	80.7 (63 - 90)	87.1 (71 - 94)
**Likelihood ratio of a Positive Test (95% CI)**	2.8 (2.2 - 3.3)	13.4 (4.540.1)	5.1 (3.7 -7.0)	-	-	-
**Likelihood ratio of a Negative Test (95% CI)**	0.33 (0.21 - 0.50)	0.45 (0.36 - 0.56)	0.28 (0.18 - 0.42)	0.14 (0.07095 - 0.2621)	0.27 (0.1967 - 0.3781)	0.18 (0.1114 - 0.2968)
**Diagnostic Odds (95% CI)**	8.4 (2.5 - 27.5)	30 (5.7 - 157.7)	18.3 (5.0- 66.1)	-	-	-
**Cohen's kappa (Unweighted) (95% CI)**	0.4 (0.2 - 0.7)	0.6 (0.35 0.8)	0.6 (0.36 0.8)	0.8 (0.4 - 1.13)	0.6 (0.2 - 0.9)	0.7 (0.3 - 1.0)

^a^ Abbreviations: CI, confidence interval; ROMA, risk of ovarian malignancy algorithm.

^b^ Data are presented as Median (Range).

**Table 5.** AUC of HE4, CA125 and the ROMA Index Patients ^a,b^

	AUC (95% CI)	Total	Group		P Value ^c^
			Benign	Malignant	
**CA125**					< 0.001
	0.828 (0.747 - 0.909)	230 ± 860	33 ± 40	486 ± 1266	
		29 (2 - 7169)	17 (2 - 140)	121 (3 - 7169)	
**HE4**					< 0.001
	0.857 (0.78 - 0.934)	146 ± 246	47 ± 19	275 ± 333	
		56 (5 - 1500)	47 (5 - 97)	114 (26 - 1500)	
**ROMA**					< 0.001
	0.907 (0.845 - 0.969)	26.7 ± 31.4	8.2 ± 5.1	50.8 ± 34.9	
		11 (1 - 99)	7.5 (1 - 22)	42 (2.5 - 99)	
**AUC, P Value**	0.026				

^a^ Abbreviations: AUC, area under the curve; ROMA, risk of ovarian malignancy algorithm.

^b^ Results are presented as mean ± SD, median (Range).

^c^ Based on Man-Whitney test.

**Table 6.** Distribution of Malignant Tumors in 4 Stages

Stage	Number	Percent of Total	Percent of Malignant
**1**	4	4.0	9.3
**2**	8	8.1	18.6
**3**	22	22.2	51.2
**4**	9	9.1	20.9

**Table 7.** Association of ROMA and Stage of Ovarian Cancer ^a^

	Early Stage	Advanced Stage
**True Positive**	9	23
**True Negative**	54	54
**False Positive**	2	2
**False Negative**	3	8
**Sensitivity**	75 (46.77 - 91.11)	74.2 (56.75 - 86.3)
**Specificity**	96.4 (87.88 - 99.02)	96.4 (87.88 - 99.02)
**Positive Predictive Value**	81.8 (52.3 - 94.86)	92 (75.03 - 97.78)
**Negative Predictive Value**	94.7 (85.63 - 98.19)	87.1 (76.55 - 93.31)
**Diagnostic Accuracy**	92.7 (83.91 - 96.82)	88.5 (80.12 - 93.64)
**Likelihood ratio of a Positive Test**	21 (7.33 - 60.17)	20.8 (7.569 - 57.02)
**Likelihood ratio of a Negative Test**	0.26 (0.1347 - 0.499)	0.27 (0.2092 - 0.3424)
**Diagnostic Odds**	81 (11.84 - 554.4)	77.6 (15.29 - 394)
**Cohen's kappa**	0.74 (0.5011 - 0.9758)	0.74 (0.5306 - 0.9456)

^a^ Data are presented as median (Range).

## 5. Discussion

Ovarian cancer is the seventh most common malignancy of female genital tract. Furthermore, it is the fifth mortality cause (2), since in 70% of cases, it progresses into advanced stage before it is diagnosed (11). Accordingly, patients’ survival chance and quality of life are increased if the disease is diagnosed in early stage and patients be referred to gyneco-oncologist before any inappropriate or insufficient therapeutic modalities are performed (12, 13). That is why many researchers try to use sonography, serum markers or other methods to be able to predict the nature of pelvic masses preoperatively and refer patients to well-equipped centers (14, 15). In that relation, the panel biomarkers of CA125 and HE4 (the ROMA index) have been selected (14, 16, 17) in the present study. These markers were checked in 99 women with ovarian masses before operation. From that number, 56 cases had benign epithelial masses, and 43 cases had malignant epithelial masses.

Regarding demographic findings in both case and control groups, the only significant difference was that members of malignant group were older than those of benign group, a fact propounded as risk factor in other studies (18). However, in contrast with previous studies, other factors, which were studied had no differences between the two groups (19, 20). As for clinical symptoms, the presence of abdominal distention during pre-diagnosis period mattered statistically. Contrary to previous studies, in the present study other clinical factors were noticed not to be associated with malignancy.

In the present study, in general the specificity, PPV and AUC of the ROMA (96.4%, 94.1%, 0.907 respectively) were higher than HE4 (91.1%, 85.7 %, 0.857, respectively) and CA125 (67.9%, 67.3%, 0.828 respectively), although except specificity (P < 0.05), the difference of other parameters was not statistically significant. Some previous studies (7, 14) have shown that measuring CA125 and HE4 (the ROMA index) together has higher DA compared to measuring each of these markers alone. Despite the fact, other studies did not show such a result (9, 21). In the present study, ROMA had a higher DA (86.9%) than HE4 (81.8%) and CA125 (75.8%) alone, although such difference was not statistically significant. As concerns the sensitivity of CA125 and HE4, in some articles the latter was shown as more sensitive than the former (7, 8, 22), and some other articles showed the vice versa (7, 10, 23). Similarly, in the present study CA125 was proved to be more sensitive than HE4 and even the ROMA in the diagnosis of epithelial ovarian cancer. Moreover, NPV of CA125 (86.4%) was more than HE4 (79.7%) and the ROMA (83.1%). Considering previous studies (24, 25), which reported different levels of CA125 and HE4 in pre-menopause and post-menopause ages, the present study divided patients into these two subgroups, which were studied separately.

In the pre-menopause group, sensitivity was similar in the ROMA and CA125 (76.2%), both of which were higher than HE4 sensitivity (57.1%). In addition, NPV and AUC of ROMA (88.9% and 0.868 respectively) were higher than HE4 (83.3 % and 0.839 respectively) and CA125 (87.2% and 0.81 respectively). None of the results were statistically significant. In this age group, the specificity, PPV and DA of ROMA (85.1%, 69.9% and 82.4% respectively) and HE4 (95.7%, 85.7% and 83.3% respectively) were higher than CA125 (72.3%, 55.2% and 73.5% respectively). Except specificity, the other two parameters were not statistically significant [the ROMA and HE4 had a higher specificity than CA125 (P ˂ 0.05)]; it may be due to small volume sampling. Such difference may become significant with more study cases, so that HE4 could be proposed in addition to CA125 to distinguish benign tumors from malignant tumors. In post-menopause group, sensitivity, NPV and DA of ROMA (81.8%, 69.2% and 87.1% respectively) were higher than HE4 (72.7%, 60% and 80.7% respectively). Moreover, PPV and specificity of all three markers were 100%. AUC of the ROMA (0.929) was higher than HE4 (0.864) and CA125 (0.924). None of these results were statistically significant in such age group.

In general, the present study examined the prognostic value of the ROMA index in patients with adnexal masses before operation, and concluded that specificity, DA and AUC of the ROMA were higher than HE4 and CA125 taken separately. Although, due to small volume sampling, gained difference was not statistically significant. The result of the present study is the same with similar previous studies (8, 23). In addition, in some studies, the ROMA level was checked to determine the prognosis and survival of patients. The present results constitute the first phase of the present study, which is performed prospectively; and it is hoped that in its continuation, more patients enter the study and more reliable results would be extracted. It is suggested to assess the relation between the ROMA index and patient’s prognosis and survival in addition to mentioned parameters. 

An important strength of this study was its cross-sectional nature, so we could calculate NPV and PPV, therefore our results can be compared with other centers results. Another point of strength of this study was that the pathologist was unaware of our study goal. Furthermore, it was the first time that such a study was performed in Iran. Our Limitation of our study was absence of multicenter data collection, and it may not be applied for other centers.
